# Lung parenchymal and cardiac appearances on computed tomography pulmonary angiography impact survival in chronic thromboembolic pulmonary hypertension: results from the ASPIRE Registry

**DOI:** 10.1183/23120541.00732-2024

**Published:** 2025-06-23

**Authors:** Lojain Abdulaal, Ahmed Maiter, Krit Dwivedi, Michael J. Sharkey, Samer Alabed, Dheyaa Alkhanfar, Alexander Rothman, Smitha Rajaram, Robin Condliffe, David G. Kiely, Andrew J. Swift

**Affiliations:** 1 Division of Cardiovascular Medicine, University of Sheffield, Sheffield, UK; 2 Department of Diagnostic Radiology, King Abdulaziz University, Jeddah, Saudi Arabia; 3 Radiology Department, Sheffield Teaching Hospitals NHS Foundation Trust, Sheffield, UK; 4 INSIGNEO, Institute for In Silico Medicine, The University of Sheffield, Sheffield, UK; 5 National Institute for Health and Care Research, Sheffield Biomedical Research Centre, Sheffield, UK; 6 Sheffield Pulmonary Vascular Disease Unit, Sheffield Teaching Hospitals NHS Foundation Trust, Sheffield, UK; 7 These authors contributed equally

## Abstract

**Background:**

Chronic thromboembolic pulmonary hypertension (CTEPH) is commonly evaluated using computed tomography pulmonary angiography (CTPA). We evaluated the frequency and impact of parenchymal and cardiac abnormalities on survival in CTEPH.

**Methods:**

Patients were identified from the ASPIRE (Assessing the Spectrum of Pulmonary Hypertension Identified at a Referral Centre) Registry. Kaplan–Meier analysis was used to assess survival.

**Results:**

290 patients (55% female, mean±sd age 65±14 years) with CTEPH were included. Mosaic perfusion was noted in 83%, lung infarction in 73% and parenchymal lung disease in 28%. The severity of mosaic perfusion and lung infarction correlated with markers of disease severity (p<0.001). Whereas the presence of mosaic perfusion was associated with improved survival in all patients (p=0.03), it did not predict outcome in those undergoing pulmonary endarterectomy (PEA) (p=0.6) and those not undergoing PEA (p=0.22). The presence of lung infarction had no impact on mortality. The presence of co-existing lung disease was associated with a worse survival (p<0.008) in patients not undergoing PEA. Mosaic perfusion was less common in patients with parenchymal lung disease (65%) compared to those without parenchymal lung disease (90%), p<0.001. An increased right/left ventricular ratio and aortic diameter predicted a worse outcome (p<0.002).

**Conclusion:**

Lung parenchymal and cardiac changes on CTPA predict outcome in CTEPH. Co-existing parenchymal lung disease is not uncommon and when present may mask the presence of mosaic perfusion. This study highlights the importance of systematically evaluating the lung parenchyma and cardiac changes in patients with CTEPH.

## Shareable abstract

**This study highlights the value of systematically evaluating vessels, cardiac chambers and the lung parenchyma in patients with CTEPH and the adverse impact of parenchymal lung disease on survival** https://bit.ly/3CV9RmU

## Introduction

Chronic thromboembolic pulmonary hypertension (CTEPH) is due to nonresolution of thrombus and is often accompanied by a microvasculopathy [1]. A large prospective study and data from the ASPIRE (Assessing the Spectrum of Pulmonary Hypertension Identified at a Referral Centre) Registry noted a 2-year incidence of approximately 2% following an acute pulmonary embolus [2, 3]. Without treatment, CTEPH is a progressive condition resulting in right heart failure and death. However, with newer approaches to treatment including pulmonary endarterectomy (PEA), balloon pulmonary angioplasty (BPA) and medical therapy, outcomes are significantly improved [1]. One of the challenges is the identification of the most appropriate therapy or therapies for treating CTEPH. Consequently, patients undergo multimodality testing, not only to characterise the extent of clot burden and estimate the severity of microvascular involvement but also assess for comorbidities, which may impact significantly on decisions to proceed to PEA [4, 5].

Computed tomography pulmonary angiography (CTPA) plays an important role in the assessment of patients with suspected and confirmed CTEPH; it is noninvasive and is recommended in international guidelines and by expert bodies for the evaluation of patients with CTEPH [6–8]. CTPA allows for an assessment of vessels providing a roadmap of the pulmonary vasculature but in addition also allows for an assessment of cardiac chambers, lung parenchyma and mediastinal structures [9]. Vascular abnormalities include attenuated vessels, stenosis, webs and eccentric thrombi [1, 10, 11]. Parenchymal abnormalities include mosaic perfusion and the sequelae of lung infarction. Lung infarction is more common in the setting of distal rather than central pulmonary emboli, as the lung peripheries receive less collateral supply from the bronchial arterial circulation [12]. Although acute infarction usually appears as areas of wedge-shaped defects over time these become organised resulting in subpleural scarring and or consolidation/cavitation [13]. Cardiac abnormalities reflecting the presence of pulmonary hypertension (PH) may be observed on CTPA and a combination of pulmonary artery (PA) diameter ≥30 mm, right ventricular (RV) outflow tract hypertrophy ≥6 mm and an RV/left ventricular (LV) ratio of ≥1 is highly predictive for the presence of PH [14].

Importantly, comorbidities impact survival in patients with CTEPH [4, 5] and can be appreciated on imaging [15]. In patients with other forms of PH such as idiopathic pulmonary artery hypertension (IPAH), even minor parenchymal lung disease on computed tomography (CT) predicts a higher mortality [16, 17]; however, the impact of these changes on outcome in patients with CTEPH is not known. The aim of this study was to evaluate the frequency of mosaic perfusion, lung infarction and lung parenchymal abnormalities on CTPA in patients with CTEPH, their correlation with disease severity and impact on survival.

## Methods

### Study participants

Patients were identified from the ASPIRE Registry between January 2008 and January 2018 [18]. The ASPIRE Registry comprises data from patients undergoing systematic evaluation for suspected PH at the Sheffield Pulmonary Vascular Disease unit including multimodality imaging and right heart catheterisation (RHC). This is a nationally designated PH referral centre serving a population of 15–20 million and adheres to annually audited standards of care. The diagnosis of CTEPH in this study was made according to international guidelines and detailed discussion by a multidisciplinary team. Adult patients (≥18 years) were eligible for inclusion in the study if they underwent CTPA in Sheffield and met guideline criteria for a diagnosis of CTEPH and patients who underwent PEA or received medical therapy. No patients who underwent BPA were included in this study; BPA was only nationally commissioned in the UK in 2018 [6]. Data on demographics, pulmonary function tests (PFT) and RHC were obtained from the ASPIRE Registry.

RHC was performed using a 7.5-F balloon-tipped thermodilution catheter (Becton-Dickinson, Franklin Lakes, New Jersey). Mean pulmonary artery pressure (mPAP) and pulmonary arterial wedge pressure (PAWP) were obtained using fluid-filled pressure. Cardiac output (CO) was assessed *via* the thermodilution method. Pulmonary vascular resistance (PVR) was calculated using the following formula: PVR=(mPAP – PAWP)/CO. The cardiac index was adjusted for body surface area (BSA) as: cardiac index=CO/BSA.

### CTPA acquisition and evaluation

CTPA studies were acquired using multidetector scanners (Toshiba Aquilion Prime and GE Medical Systems) with standard acquisition parameters, as follows: tube current 80–700 mA with automatic dose reduction tube voltage 120 kV, pitch 1, slice thickness 0.5 mm×80 mm, rotation speed 0.275. 60 mL of intravenous iodinated contrast (Omnipaque 350, GE Healthcare) was injected at a rate of 5 mL·s^−1^. Bolus tracking was performed over the PA using a manual fast start, with acquisition triggered over a threshold of 220 HU. Patients were excluded from the study if their CTPA images were considered to be suboptimal; for example, where contrast opacification of the pulmonary arteries was poor or there was significant movement artefact. This choice was taken during the image analysis stage, which evaluated the quality and readability of the CTPA images. Data on PEA status was collected from patient records and surgical databases at the time of census.

**FIGURE 1:**
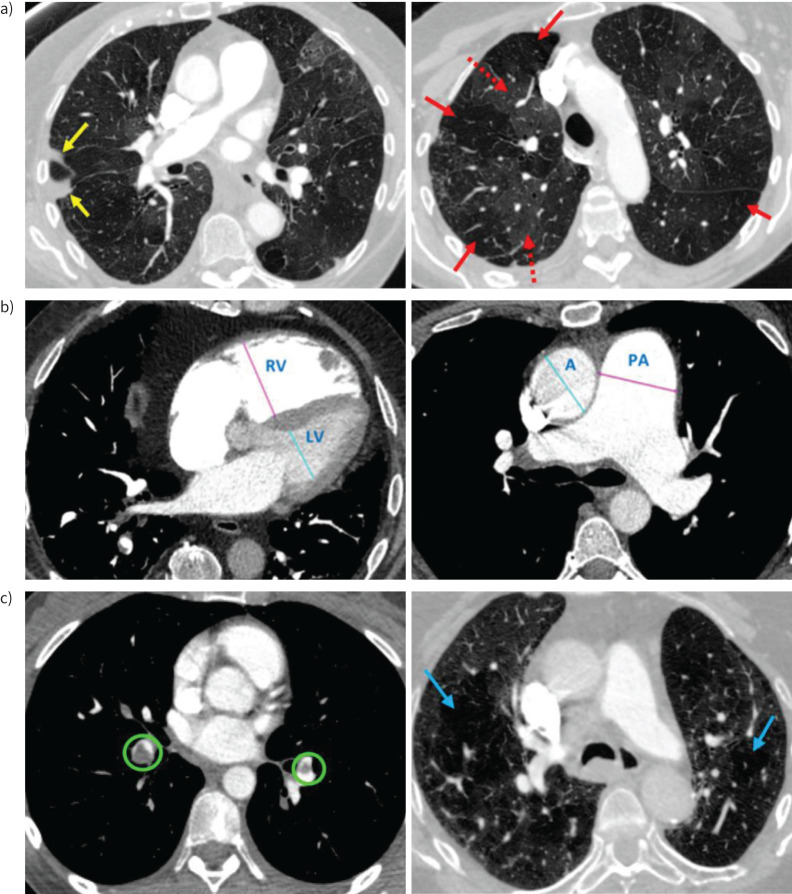
a) Examples of lung parenchymal features of chronic thromboembolic pulmonary hypertension (CTEPH) on computed tomography pulmonary angiography (CTPA). Axial CTPA images with standard lung windows from two different patients. The patient on the left showed lung infarction within the right middle lobe (yellow arrows). This typically appears as focal wedge-shaped consolidation at the lung periphery which resolves over time. Both patients exhibit mosaic perfusion. This refers to regional hyperattenuation (dashed red arrows) and hypoattenuation (solid red arrows) of the lung parenchyma, of which abnormal lung perfusion is a cause. It is important to note that neither lung infarction nor mosaic perfusion are specific to CTEPH and there is a broad differential diagnosis for both of these imaging findings. b) Examples of cardiac changes commonly seen in CTEPH on CTPA. Two axial CTPA images with standard soft tissue windows are shown. The right image shows marked dilatation of the right ventricle (RV) relative to the left ventricle (LV), with an RV/LV ratio of > 1.0, consistent with RV dysfunction. The left image shows dilatation of the pulmonary artery (PA) trunk relative to the ascending aorta (AA), with a PA/AA ratio of >1.0. c) Examples of CTEPH within left and right segmental disease (green circles), and emphysema lung disease in both lungs (blue arrows).

A semi-quantitative approach was used to evaluate CTPA images, with visual assessment being used as a tool to identify and classify abnormalities. CTPA studies were evaluated by a single consultant cardiothoracic radiologist (A.S., 13 years of experience) blinded to clinical parameters. The images were evaluated on axial slices using standard lung (level −362 HU and width 1324 HU) and soft tissue (level 40 HU and width 440 HU) windows. A standardised scoring method was used to categorise lung parenchymal disease, ensuring consistency, as follows: 0 indicating no disease, 1 indicating minor disease (1–25%), 2 indicating mild disease (26–50%) and 3 indicating moderate-to-severe disease (>50%). The evaluation focused on the following features, each classified as nil, minor, mild or moderate-to-severe, as follows: 1) appearances of the lung parenchyma, including mosaic perfusion patterns, lung infarction and any coexisting lung abnormalities (such as fibrosis, emphysema, or other lung disease); 2) vascular appearances, including the presence of CTEPH features (*e.g.* eccentric thrombus, strands or webs within pulmonary arteries), the distribution of thromboembolic disease (central/segmental/distal) and bronchial artery appearances (unopacified/normal/prominent/dilated); 3) technical adequacy, including the presence of artefacts. Cardiac chamber and vessel measurements were assessed by one radiographer (L.A., 4 years of cardiac CT experience). All measurements were made in the axial plane following training by A.S. as previously described [9]. The maximal diameters of the ascending aorta (AA) and PA trunk were measured at the level of the PA bifurcation. The diameter of the right ventricle (RV) and left ventricle (LV) were measured on the slices at which they appeared maximal. The PA/AO and RV/LV ratios were subsequently calculated (figure 1).

### Statistical analysis

Continuous data are reported as the mean±sd or median with interquartile range (IQR). Categorical data are reported as percentages and frequency. Pearson's correlation coefficient was used to evaluate the association between two continuous variables, specifically dichotomised mosaic perfusion and infarction data (grouped as 0 and 1). Group comparisons were conducted on mosaic perfusion and infarction data compared to pulmonary haemodynamic parameters, which were categorised into four levels (nil, minor, mild and moderate/severe), using a one-way ANOVA followed by *post hoc* Bonferroni correction. Kaplan–Meier analysis was used to determine the relationship between various CT parenchyma characteristics and survival after PEA treatment, with the log-rank test used to compare survival curves. Cox proportional hazards regression was used to assess the prognostic importance of CT measurements and lung parenchymal features. SPSS Statistics (SPSS version 27, IBM) and GraphPad Prism (v8; GraphPad, La Jolla, CA, USA) were used to conduct all statistical analyses, with a significance threshold of p<0.05.

As part of the national service specification for patients with pulmonary hypertension, patients receiving treatment should undergo regular assessment. There were no patients lost to follow-up during the duration of this study. Mortality data was obtained from the National Health Service Personal Demographics Service. The census date for the study was 10 January 2022. This study received ethical approval through the ASPIRE Registry (16/YH/0352).

## Results

### Study population

**FIGURE 2:**
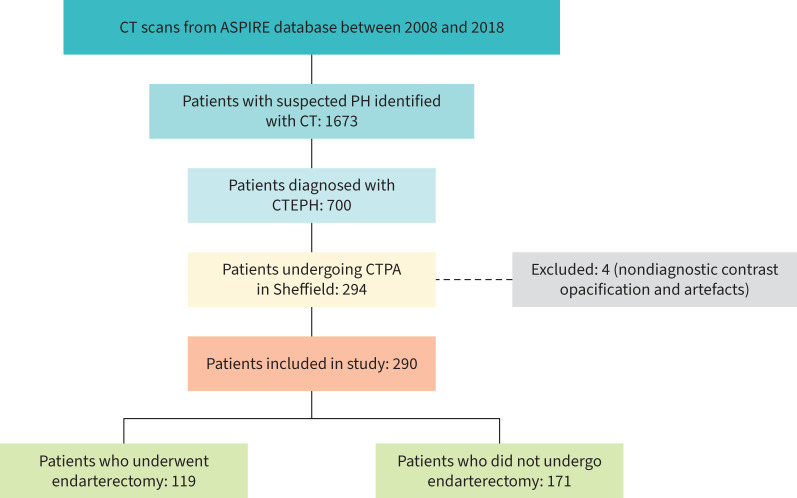
Study flow diagram. CT: computed tomography; ASPIRE: Assessing the Spectrum of Pulmonary Hypertension Identified at a Referral Centre; PH: pulmonary hypertension; CTEPH: chronic thromboembolic pulmonary hypertension; CTPA: computed tomography pulmonary angiography.

Between 2008 and 2018, 700 patients were diagnosed with CTEPH in Sheffield and 294 underwent CTPA there. Four patients were excluded from the study due to poor image quality leaving 290 patients for analysis (figure 2). Of these, 272 patients (93.7%) had available RHC data and 260 patients (89.6%) had available PFT data.

The mean±sd age was 65±14 years and 55% were female (table 1). mPAP was 45±12 mmHg and PVR 645±414 dyn.s.cm**^−^**^5^. 25 patients (9%) were in World Health Organization functional class (WHO FC) II, 231 (79%) in WHO FC III and 30 (10%) in WHO FC IV.

**TABLE 1:** Characteristics of patients undergoing right heart catheterisation and pulmonary function tests, with a comparison between patients who underwent pulmonary endarterectomy (PEA) and those who did not (no PEA)

Patient characteristics	Full cohort n=290	Endarterectomy n=119 (41%)	No endarterectomy n=171 (59%)	p-value
**Age (years)**	65±14	60±14	67±14	<0.001
**Sex (female)**	158 (54.5%)	68 (57%)	90 (53%)	0.44
**WHO FC**	II (25), III (231), IV (30)	II (6), III (101), IV (10)	II (19), III (130), IV (20)	0.47

Right heart catheterisation	n=272 (94%)	n=112 (94%)	n=160 (94%)	p-value
**PVR (dyn^.^s^.^cm^−5^)**	646±414	666±424	631±409	0.5
**mPAP (mmHg)**	45±12	45±12	45±12	0.99
**PAWP (mmHg)**	12.6±5.1	12.6±4.7	12.7±5.4	0.72
***S*_vO_2__ (%)**	62±9	63±8	61±9.1	0.16
***S*_aO_2__ (%)**	94±3.8	93±4.1	94±3.4	0.3
**Cardiac index (L·min^−1^·m^−2^)**	2.6±0.8	2.5±0.8	2.6±0.9	0.22
**Cardiac output (L·min^−1^)**	4.7±1.6	4.6±1.5	4.9±1.8	0.12

Pulmonary function test	n=260 (90%)	n=114 (96%)	n=146 (85%)	p-value
**Predicted FEV_1_ (%)**	76.3±20.3	80.1±19.06	73.3±20.8	0.008
**Predicted FVC (%)**	88.4±21.5	91.8±20.07	85.8±22.2	0.02
**Predicted *T*_LCO_ (%)**	60.2±18.3	62±15.8	59±20	0.17

Data are presented as mean±sd or counts (percentage). Haemodynamic results include pulmonary vascular resistance (PVR), mean pulmonary arterial pressure (mPAP), pulmonary artery wedge pressure (PAWP), venous oxygen saturation (*S*_vO_2__), oxygen saturation (*S*_aO_2__), cardiac index and cardiac output. Pulmonary function results include predicted forced expiratory volume in 1 s (FEV_1_), predicted forced vital capacity (FVC) and predicted transfer factor of the lung for carbon monoxide (*T*_LCO_). p-values (p<0.05) indicate significant differences between the pulmonary endarterectomy and no pulmonary endarterectomy groups. WHO FC: World Health Organization functional class.

PEA was performed in 119 patients (41%) with a median duration of 7 months (IQR 0–11) to surgery.

### CTPA findings

#### Distribution of CTEPH

Of patients with CTEPH, 25% had central or lobar disease, 61% segmental disease and 14% subsegmental disease. The distribution of CTEPH did not correlate with either extent of lung infarction (r=−0.07, p=0.18) or mosaic perfusion (r=−0.03, p=0.61).

#### Mosaic perfusion pattern and lung infarction

Mosaic perfusion and lung infarction were present in 83% and 73% of patients, respectively, with both present in 69% (table 2). The severity of mosaic perfusion showed a significant correlation with RV/LV ratio (r=0.22, p<0.001). There was no significant correlation found between lung infarction and RV/LV ratio (r=0.06, p=0.29). No correlation was found between mosaic perfusion or lung infarction with PA/AO ratio (r=0.01, p=0.73 and r=−0.02, p=0.79, respectively).

**TABLE 2:** Findings on computed tomography pulmonary angiography (CTPA) images in patients with chronic thromboembolic pulmonary hypertension (CTEPH)

Variables	Full cohort n=290	Endarterectomy n=119 (41%)	No endarterectomy n=171 (59%)	p-value
**Central CTEPH**	73 (25%)	26 (22%)	46 (27%)	0.4
**Segmental CTEPH**	177 (61%)	75 (63%)	102 (60%)	0.64
**Subsegmental CTEPH**	40 (14%)	18 (15%)	22 (13%)	0.7
**Mosaic perfusion**	241 (83%)	105 (88%)	136 (80%)	0.07
**Lung infarction**	211 (73%)	90 (76%)	121 (71%)	0.43
**Abnormal bronchial artery**	228 (78%)	90 (76%)	138 (81%)	0.37
**Lung disease (emphysema/fibrosis/ other)**	81 (28%) (45/11/25) (16%/4%/9%)	25 (21%) (17/3/5) (14%/3%/4%)	56 (33%) (27/7/22) (16%/4%/13%)	0.03
**Lung cavity**	11 (3.8%)	6 (5%)	5 (3%)	0.36
**Coronary artery disease**	189 (65%)	69 (58%)	120 (70%)	0.03
**Kidney disease**	21 (7%)	5 (4%)	16 (9%)	0.1
**Calcific aortic valve**	24 (8%)	7 (6%)	17 (10%)	0.28
**PA diameter (cm)**	3.4±0.49	3.4±0.55	3.4±0.44	0.85
**AO (cm)**	3.26±0.44	3.17±0.44	3.3±0.43	0.007
**RV (cm)**	4.46±0.68	4.32±0.7	4.57±0.67	0.003
**LV (cm)**	3.52±0.68	3.6±0.68	3.48±0.69	0.16
**PA/AO ratio**	1.05±0.17	1.08±0.17	1.03±0.17	0.03
**RV/LV ratio**	1.31±0.34	1.25±0.33	1.36±0.34	0.005

Data are mean values±sd or counts (percentage). p-values (p<0.05) indicate significant differences between the pulmonary endarterectomy and no pulmonary endarterectomy groups. AO: aorta; LV: left ventricle; PA: pulmonary artery; RV: right ventricle.

#### Bronchial circulation

Dilated bronchial arteries were found in 78% of patients. Bronchial artery dilatation showed a weak statistical association with mosaic perfusion (r=0.12, p=0.035). Bronchial artery dilatation showed no statistical association with infarction (r=0.02, p=0.72).

No statistically significant correlation was identified for either age or sex with different chronic pulmonary embolism distributions, mosaic perfusion, bronchial artery dilatation or PA diameter.

#### Parenchymal lung disease

81 of 290 patients (28%) had co-existing parenchymal lung disease. Of the total cohort of CTEPH, emphysema was present in 45 (15.5%), interstitial lung disease in 11 (3.8%) and other parenchymal abnormalities in 25 (9%) including air trapping in seven (2.4%), consolidation in four (1.4%), ground glass in two (0.7%), bronchiectasis in six (2.1%), lung cyst in two (0.7%) and pleural involvement (pleural plaques/effusion) in four (1.4%).

Mosaic perfusion was present in 53 out of 81 (65%) of patients with parenchymal lung disease compared to 188 out of 209 (90%) of patients without parenchymal lung disease, p<0.001. Lung infarction was also less common in patients with parenchymal lung disease and was present in 45 out of 81 patients (56%) compared to 166 out of 209, (79%) of patients without parenchymal lung disease, p<0.001.

#### Extent of lung parenchymal abnormalities on CTPA and RHC measurements

RHC was performed at a median of 0 days (IQR 0–1 days) around CTPA. The extent of mosaic perfusion and lung infarction correlated positively with PVR (r=0.32, p<0.001 and r=0.24, p<0.001) and negatively with venous oxygen saturation (*S*_vO_2__) (r=−0.31, p<0.001 and r=−0.24, p<0.001). Whilst the extent of mosaic perfusion (r=0.20, p<0.001) correlated with mPAP there was no correlation between infarction severity (r=0.07, p=0.20) and mPAP.

**FIGURE 3:**
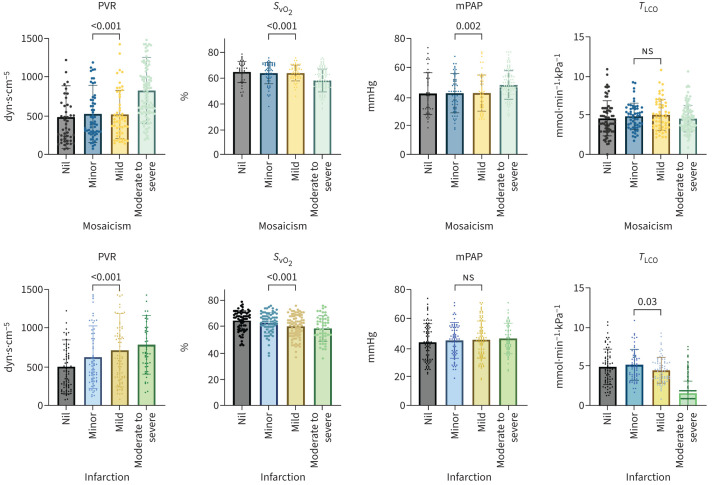
Group comparison of the extent of lung mosaic perfusion (top) and lung infarction (bottom) with right heart catheterisation and *T*_LCO_ using one-way ANOVA. Pulmonary vascular resistance (PVR), mean pulmonary arterial pressure (mPAP), venous oxygen saturation (*S*_vO_2__), and transfer factor of the lung for carbon monoxide (*T*_LCO_). NS: nonsignificant.

Figure 3 presents the one-way ANOVA analysis of the severity levels of mosaic perfusion and lung infarction.

#### The extent of lung parenchymal features on CTPA and spirometry and gas transfer measurements

There was no correlation between the extent of mosaic perfusion with forced expiratory volume in 1 s (FEV_1_) or forced vital capacity (FVC) (r=−0.1, p=0.1 and r=−0.05, p=0.4). The extent of infarction showed a weak negative correlation with FEV_1_ (r=−0.13, p=0.02) but not with FVC (r=−0.07, p=0.25).

There was no correlation between the extent of mosaic perfusion and transfer factor of the lung for carbon monoxide (*T*_LCO_) whereas the extent of infarction correlated negatively (r=−0.15, p=0.015) with *T*_LCO_.

#### Kaplan–Meier survival and Cox regression analysis for parenchymal, vascular and cardiac CT features

For all patients, the presence of mosaic perfusion was associated with improved survival with 1- and 3-year survival of 90 and 77% compared to 91 and 65%, in patients without mosaic perfusion, respectively, p=0.03. In contrast, the presence of lung infarction had no impact on mortality. For all patients, the presence of parenchymal lung disease was associated with a worse outcome with 1- and 3-year survival of 81 and 61% compared to 93 and 79% in patients without parenchymal disease, respectively, p=0.004. For patients undergoing PEA, there was no significant difference in survival rates at 1 year (97 *versus* 96%), 3 years (96 *versus* 88%) and 5 years (89 *versus* 82%) between those with and without parenchymal lung disease, respectively (p=0.98). For patients not undergoing PEA, the presence of parenchymal lung disease had lower survival rates at 1 year (73 *versus* 91%), 3 years (48 *versus* 72%) and 5 years (41 *versus* 57%) compared to patients without parenchymal lung disease, respectively (p=0.008).

**FIGURE 4:**
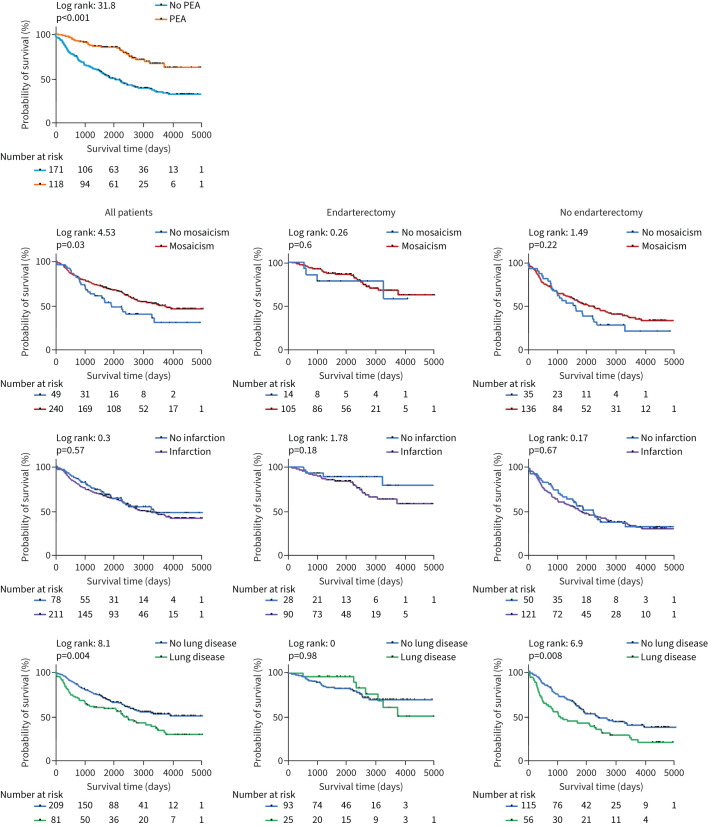
First Kaplan–Meier survival curve comparing chronic thromboembolic pulmonary hypertension patients who underwent pulmonary endarterectomy (PEA) to those who did not. along with additional curves predicting mosaic perfusion, infarction and lung disease related mortality for patients who had PEA and who did not undergo PEA after computed tomography (CT). These curves are based on CT scan findings and show numbers at risk by years.

**FIGURE 5:**
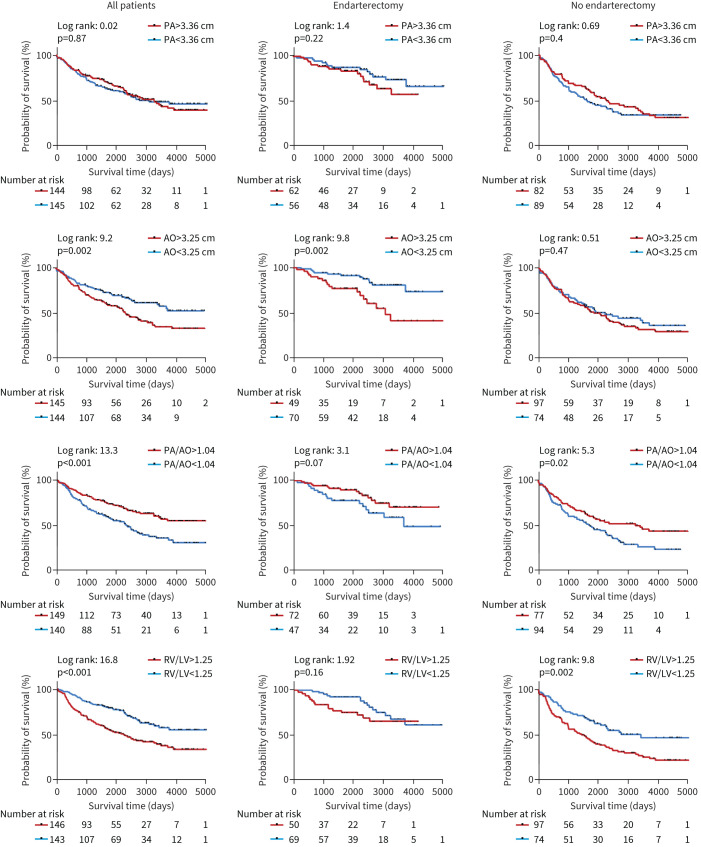
Kaplan–Meier survival curves examining the impact of computed tomography pulmonary angiography measured vessels and cardiac chambers on survival in all patients with chronic thromboembolic pulmonary hypertension, those undergoing pulmonary endarterectomy (PEA) and those not undergoing PEA (pulmonary artery (PA), aorta (AO), PA/AO ratio and right ventricle (RV)/left ventricle (LV) ratio).

Kaplan–Meier curves examining the impact of mosaic perfusion, lung infarction, co-existing lung disease and CT measurements on survival in all patients, those undergoing PEA and those not undergoing PEA are presented in figures 4 and 5. Characteristics of patients and Cox regression analysis of major vessels and cardiac chambers for patients undergoing and not undergoing PEA are shown in tables 2 and 3.

**TABLE 3:** Univariate and multivariate cox-regression analysis for computed tomography assessed vessels, cardiac changers and parenchymal change in patients undergoing and not undergoing pulmonary endarterectomy

Variables	Univariate analysis			Multivariate analysis^#^		
Endarterectomy (n=119)	HR	95% CI	p-value	HR	95% CI	p-value
**PA diameter (cm)**	1.48	0.79–2.79	0.22	NA		
**AO (cm)**	8.33	3.38–20.5	<0.001	6.93	2.5–18.6	<0.001
**PA/AO ratio**	0.07	0.006–0.9	0.04	NA		
**RV (cm)**	1.53	0.92–2.5	0.09	NA		
**LV (cm)**	0.93	0.56–1.5	0.7	NA		
**RV/LV ratio**	1.7	0.64–4.9	0.26	NA		
**Mosaic perfusion**	0.76	0.26–2.18	0.6	NA		
**Infarction**	2.02	0.7–5.8	0.19	NA		
**Lung disease**	1	0.43–2.3	0.9	NA		

No endarterectomy (n=171)	HR	95% CI	p-value	HR	95% CI	p-value
**PA diameter (cm)**	0.76	0.4–1.19	0.24	NA		
**AO (cm)**	1.29	0.85–1.96	0.22	NA		
**PA/AO ratio**	0.29	0.09–0.93	0.03	NA		
**RV (cm)**	1.51	1.14–2.02	0.004	NA		
**LV (cm)**	0.6	0.45–0.85	0.003	NA		
**RV/LV ratio**	3.59	2.08–6.2	<0.001	3.9	2.2–6.9	<0.001
**Mosaic perfusion**	0.75	0.48–1.18	0.22	NA		
**Infarction**	1.09	0.71–1.68	0.67	NA		
**Lung disease**	1.68	1.13–2.4	0.009	1.79	1.1–2.7	0.005

^#^: Multivariate including significant univariate predictors and with adjustment for age, sex and pulmonary vascular resistance. AO: aorta; HR: hazard ratio; LV: left ventricle; NA: not applicable; PA: pulmonary artery; RV: right ventricle.

## Discussion

In this study we have shown that the presence of parenchymal lung changes on CTPA is common in CTEPH and we have demonstrated for the first time that it impacts negatively on survival. In addition, to our knowledge we have shown for the first time that an increase in aortic diameter predicts worse outcomes, likely due to the impact of underlying comorbidities. In addition, measurements of vessels and cardiac chambers have prognostic value in CTEPH. We have also confirmed that mosaic perfusion and lung infarction are common features of CTEPH and correlate albeit weakly with indicators of disease severity. This study highlights the value of systematically evaluating vessels, cardiac chambers and the lung parenchyma in patients with CTEPH and the adverse impact of parenchymal lung disease on survival.

Despite a number of registries reporting on the frequency of co-morbidities in patients with CTEPH [4, 5], there is relatively limited published data on CT parenchymal lung disease in patients with CTEPH and their correlation with outcome. A key finding from this study is the high prevalence of co-existing lung disease (28%) noted on imaging in patients with CTEPH including the presence of emphysema in 15.5% and interstitial lung disease in 3.8%. This is higher than the prevalence of COPD (9.5%) and interstitial lung disease (1.3%) noted in the first International CTEPH Registry [4]. In patients who meet guideline criteria for IPAH the presence of even minor degrees of lung disease impact negatively on survival [16]. In our study, moderate to severe lung disease was associated with the poorest mortality outcomes in both the PEA and non-PEA patients. The absence of patients with severe lung diseases from PEA eligibility is possibly the reason for the increased incidence and severity of parenchymal abnormalities seen in the non-PEA group. As a result, PEA patients with parenchymal abnormalities commonly present with less severe lung disease. However, regardless of PEA intervention, the extent and severity of parenchymal abnormalities remain critical in determining the prognosis of the patient. This is thought to reflect environmental factors, such as smoking, and their impact on the pattern of pulmonary vascular involvement [19]. In our study, the presence of lung disease was associated with a worse outcome in patients with CTEPH with the exception of patients undergoing surgery. However, in the surgical population, those with more significant lung disease were excluded due to concerns of increased risk of peri-operative mortality and this is reflected in significantly higher FEV_1_ and FVC in those undergoing surgery compared to those who did not. Previous studies in patients with CTEPH have identified a number of other comorbidities such as malignancy and chronic kidney disease that were independent predictors of a worse outcome in patients with CTEPH [5]. However, the adverse impact of parenchymal lung disease on outcome in CTEPH, has not to our knowledge, been previously reported. For patients with CTEPH, associated CT findings of parenchymal lung disease may reflect additional deleterious pulmonary vascular involvement and loss of the capillary vascular bed. This form of pulmonary vascular involvement may explain why such patients have a worse prognosis. This study highlights the importance of identifying parenchymal lung disease given the negative impact it has on survival.

Our study confirms the finding of previous studies that features such as mosaic perfusion and lung infarction are common in patients with CTEPH. Previous studies have shown significant correlations between the presence of mosaic perfusion and key indicators of disease severity in pulmonary hypertension. Haemodynamic markers such as PVR and *T*_LCO_ have prognostic value in various forms of pulmonary hypertension, including CTEPH [5, 20, 21]. In this study, the extent of mosaic perfusion and lung infarction correlated positively with PVR and negatively with *S*_vO_2__, consistent with the observation that these imaging characteristics reflect more severe pulmonary vascular disease. Patients with lung infarction compared to those without infarction also had a significantly lower *T*_LCO_, whereas the presence or absence of mosaic perfusion did not impact on *T*_LCO_. In the present study lower *T*_LCO_ was also associated with the presence of co-existing lung disease. We identified 11 patients with lung cavities (3.8%); the presence of a lung cavity was not associated with mortality.

Several studies have explored the correlation between mosaic perfusion and haemodynamic parameters in CTEPH. A study found that in 145 patients with CTEPH, both mPAP and PVR demonstrated a significant correlation with the degree of mosaic perfusion [22]. Another study showed that in 27 patients with CTEPH, 25 of whom had RHC data, mosaic perfusion had a significant positive correlation with PVR but no correlation with mPAP [23]. Our study showed a significant though modest correlation between mosaic perfusion with mPAP, PVR and *S*_vO_2__. In addition to confirming the association of mosaic perfusion with disease severity, we have also shown that lung infarction is associated with more severe haemodynamic disease but also abnormalities of gas exchange, reflected in a lower *T*_LCO_.

It has been noted that patients with CTEPH without mosaic perfusion exhibit a higher mortality than patients with mosaic perfusion, although the rationale for this is not clear. We postulate this may in part be due to the higher incidence of co-existing parenchymal lung disease in patients without mosaic perfusion. In addition to the association of lung disease with more widespread vascular damage, parenchymal lung disease by impacting on lung attenuation may mask the detection of mosaic perfusion. Our findings suggest that for patients undergoing PEA, the presence of mosaic perfusion or infarction does not significantly impact all-cause mortality. While in patients not undergoing PEA, the absence of mosaic perfusion is associated with increased mortality, likely relating to the higher proportion of patients with parenchymal lung disease in this group. However, in patients without lung disease, the extent of mosaic perfusion did not predict mortality. These findings highlight the importance of comprehensively assessing both vascular and parenchymal abnormalities in patients with CTEPH.

Finally, we have shown that CTPA features of vessels and cardiac chambers have prognostic value in patients with CTEPH. In particular, we have shown that an increase in RV diameter and an increase in RV/LV ratio predict a worse outcome in patients with CTEPH regardless of whether patients undergo or do not undergo PEA. The adverse prognostic impact of an increased RV/LV ratio has been demonstrated in other forms of PH, but to our knowledge it is the first time it has been observed in a large cohort of patients with CTEPH [24, 25]. It is known that more severe haemodynamic disease, characterised by severe increases in PVR identify patients with CTEPH at high risk of surgical intervention [26]; whether an elevation for RV/LV ratio over a particular threshold could also be used to identify a high-risk group requires further exploration. We have also shown that an increased aortic diameter predicts a worse outcome in CTEPH regardless of whether patients underwent PEA. An increase in aortic diameter occurs with increasing age but is also a marker of other comorbidities such as hypertension. In CTEPH, a large left atrium (frequently the sequelae of systemic hypertension) is a risk factor for a worse outcome following PEA [15]. Lower PA/aorta ratio was found to be a risk factor for worse outcome in those not undergoing endarterectomy, we suspect this may again be driven by the relative increase in aortic diameter, though further studies are required to confirm. Given that left heart disease may be unmasked following PEA and the left atrial size and pulmonary arterial wedge pressure measurement may be less reliable markers of left heart disease in the presence of high right-sided pressures, further exploration of the aortic diameter as a risk factor in patients with CTEPH requires further study.

There are a number of limitations in this study. Patients were retrospectively identified from a single centre and a number of the CT assessments are qualitative and were read by a single experienced radiologist. Further work to evaluate the utility of imaging derived lung patterns for prediction of outcomes following BPA is required. Surgical resection rates were 41% for this population similar to the 43% in the UK as reported in the 14th UK National Audit of pulmonary hypertension of 4103 patients with CTEPH undergoing evaluation; suggesting that this population is representative of the UK CTEPH population.

In conclusion, systematic assessment of patients with CTEPH undergoing CTPA highlights that mosaic perfusion, lung infarction and parenchymal lung disease are commonly observed. Parenchymal lung disease and CT features of more severe pulmonary hypertension impact negatively on survival regardless of whether patients undergo PEA and should be considered when counselling patients.

## Supplementary material

**Please note:** supplementary material is not edited by the Editorial Office, and is uploaded as it has been supplied by the author. Supplementary material 00732-2024.SUPPLEMENT
