# Supplementary material for: Lung parenchymal and cardiac appearances on computed tomography pulmonary angiography impact survival in chronic thromboembolic pulmonary hypertension: results from the ASPIRE Registry
Source: ERJ Open Res. 2025 Jun 23;11(3):00732-2024. doi: 10.1183/23120541.00732-2024 (PMC12183745; doi:10.1183/23120541.00732-2024)
Supplement: Supplementary file 1 [file 00732-2024.SUPPLEMENT.pdf]

Supplementary material

CT features	mPAP	PVR	SVO2	TLCO	RV/LV ratio
Mosaic perfusion	0.002	<0.001	<0.001	NS	<0.001
Infarction	NS	<0.001	<0.001	0.03	NS
Lung disease	NS	NS	NS	0.002	NS
Clot location	0.041	0.034	<0.001	NS	NS
Bronchial arteries	0.03	0.004	<0.001	NS	<0.001

Table 1. One-way ANOVA with post-hoc Bonferroni test to identify significant mean differences between groups. The values presented in the table are p-values, indicating the level of statistical significance for each comparison between groups.

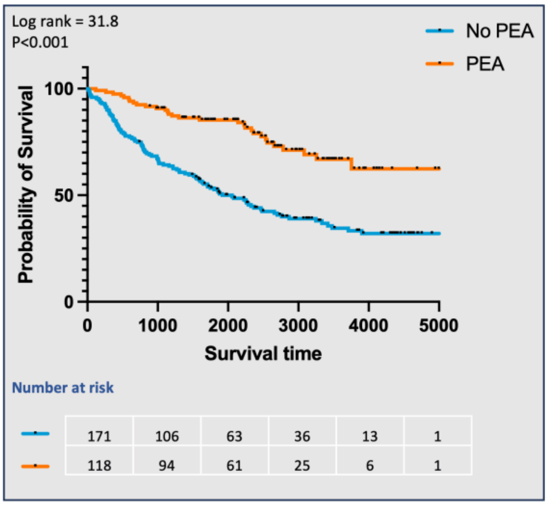

Figure1. Kaplan-Meier survival curve comparing survival outcomes in CTEPH patients who underwent pulmonary endarterectomy (PEA) versus those who did not, for the full cohort.
